# Competence in metered dose inhaler technique among community pharmacy professionals in Gondar town, Northwest Ethiopia: Knowledge and skill gap analysis

**DOI:** 10.1371/journal.pone.0188360

**Published:** 2017-11-27

**Authors:** Sewunet Admasu Belachew, Fasil Tilahun, Tirsit Ketsela, Asnakew Achaw Ayele, Adeladlew Kassie Netere, Amanual Getnet Mersha, Tamrat Befekadu Abebe, Begashaw Melaku Gebresillassie, Henok Getachew Tegegn, Daniel Asfaw Erku

**Affiliations:** 1 Department of Clinical Pharmacy, School of Pharmacy, University of Gondar Chechela Street, Lideta Sub city Kebele, Gondar, Ethiopia; 2 Department of Gynecology and obstetrics, School of Medicine, University of Gondar Chechela Street, Lideta Sub city Kebele, Gondar, Ethiopia; The Chinese University of Hong Kong, HONG KONG

## Abstract

**Background:**

When compared to systemic administration, if used correctly inhalers deliver a smaller enough percent of the drug right to the site of action in the lungs, with a faster onset of effect and with reduced systemic availability that minimizes adverse effects. However, the health professionals' and patients' use of metered dose inhaler is poor.

**Objective:**

This study was aimed to explore community pharmacy professionals' (pharmacists' and druggists') competency on metered dose inhaler (MDI) technique.

**Method:**

A cross sectional study was employed on pharmacy professionals working in community drug retail outlets in Gondar town, northwest Ethiopia from March to May 2017. Evaluation tool was originally taken and adapted from the National Asthma Education and Prevention Programmes of America (NAEPP) step criteria for the demonstration of a metered dose inhaler to score the knowledge/proficiency of using the inhaler.

**Result:**

Among 70 community pharmacy professionals approached, 62 (32 pharmacists and 30 druggists/Pharmacy technicians) completed the survey with a response rate of 85.6%. Only three (4.8%) respondents were competent by demonstrating the vital steps correctly. Overall, only 13 participants got score seven or above, but most of them had missed the essential steps which included steps 1, 2, 5, 6, 7 or 8. There was a significant difference (*P* = 0.015) in competency of demonstrating adequate inhalational technique among respondents who took training on basic inhalational techniques and who did not.

**Conclusion:**

This study shown that, community pharmacy professionals' competency of MDI technique was very poor. So as to better incorporate community pharmacies into future asthma illness management and optimize the contribution of pharmacists, interventions would emphasis to improve the total competence of community pharmacy professionals through establishing and providing regular educational programs.

## Background

Asthma is a chronic inflammatory condition of the airways that affects roughly 358 million peoples [1]. It is a terrifying universal health problem with an increasing prevalence worldwide. Worldwide, approximately 300 million peoples have asthma and 10% adult population over the age of 40 years may have a diagnosis of chronic obstructive pulmonary disease (COPD) [2]. All age groups are affected by this chronic airway disease with higher burden of disability [3]. Short-acting beta2 agonists are the front line management [4]. Currently, corticosteroids are the most effective treatment available for long-term asthma control.

Inhaled forms of corticosteroids are typically used in the long-term management [5]. Metered dose inhaler (MDI) device is usually used, proper inhaler technique and adequate adherence are indispensable. Concerning the technique, explicit steps and excellent harmonization are required for the appropriate use of this device.

Currently, beclomethasone and salbutamol inhalations are obtainable in all private, state procurement center and hospital pharmacies in Ethiopia. Poor asthma management is majorly as a result of miss diagnosis and improper or inadequate treatment [6]. Thus, good level of adherence to these medications is the cornerstone in the long-standing management of asthma as non-adherence or inhaler mishandling increases mortality, morbidity, and hospital encounter [7, 8]. Studies described that around 90% of asthma and COPD victims miss use their inhalers [9, 10]. Therefore, patients prescribed with MDI should be appropriately educated by experts about inhaler technique as it will lead to improved adherence and management of the disease [11]. Community pharmacists are an indispensable part of healthcare taskforce in patient counselling.

However, in most studies conducted around the globe, pharmacists have suboptimal knowledge and skill on MDI technique. A recent published article emphasized that merely 7% of healthcare providers possibly will demonstrate all the proper steps of MDI use [12]. In 2014, the National Review of Asthma Deaths (NRAD) pointed out that low level of understanding and incorrect use of inhalers was assumed to have contributed to a huge figure of the 195 asthma deaths [13]. Incorrect asthma inhaler device use is linked with poor asthma control and more frequent emergency department visits and it is allied with many risk factors including poor education/instruction of patient [14].

Many papers found comparable results with doctors, nurses and pharmacists having poor knowledge on the optimal use of different inhalers [15]. Appropriate MDI use experience among clients and medical experts is still defective [16].

Another simulated study done with the aim to assess pharmacists' skill in making proper use of MDI inhalers shown a result that overall pharmacists had poor recognition in properly addressing all the steps while using inhaler. In addition, explored that pharmacy professionals’ age status could influence their level of knowledge in addition to their job experience which was found to increase their level of knowledge towards using the inhaler correctly [17].

A study done in Nepal on nurses, physicians and pharmacists concluded as healthcare professionals MDI use were poor before intervention and intervention has took the lion share in changing the existing trend to a better practice [18]. A simulated client based study done in Nigeria generalized that community pharmacists lack the knowledge and skill of demonstrating basic steps in proper use of MDI [19]. In a study done in Mekelle, Ethiopia, MDI technique of pharmacy professionals was very limited [20].

The National Institute for Health and Care Excellence (NICE) guideline for people with COPD and the British Thoracic Society guideline for Asthma suggested sufficient training and education on correct use of inhaler to patients prior to providing the MDI [21].

It may not be shocking that patients use inhalers wrongly, this is because professionals understanding of the right use of these devices is also poor. Therefore, it is highly suggested to frequently assess health care experts’ competency in demonstrating proper MDI use with final goal of improving inhalational treatment outcomes [15]. Although Gondar has many drug stores and pharmacies with many professionals [22], this paper is pioneer to explore the competence of community pharmacy professionals’ in correctly demonstrating MDI use in Gondar town, north western Ethiopia.

As there are no studies published in Gondar town and Amhara regional state as a whole, this study will add to the existing literature gap in the area of competence of community pharmacy professionals’ on MDI. Furthermore, the findings will inform many stakeholders including governmental, nongovernmental organizations and academic institutions about potential gaps and barriers to patient counseling for a better practice.

## Method

### Study setting and design

An interview based cross-sectional survey was conducted to evaluate community pharmacy professionals' competence on MDI technique. It was done in Gondar town, which is located about 750 Km Northwest of Addis Ababa. As to the 2007 Ethiopian population and housing census report, the town had an estimated population of 206,987.

Gondar town has 42 community pharmacies and 20 drug stores. The study was undertaken from March to May 2017. All community pharmacy professionals (CPPs) who were working in Gondar town community pharmacies and drug stores during the study period were targeted.

### Sample size and sampling procedure

Simple convenience sampling technique was used and a total 62 community pharmacy professionals (pharmacists and druggists/Pharmacy Technicians) were included in this study.

### Data collection technique and management

Evaluation (data collection) tool was adapted from the National Asthma Education and Prevention Programs of America (NAEPP) step criteria for demonstration of a MDI to score the competency of use of MDIs by health care providers [23], and checked for suitability (Each steps with respect to culture, religion and other affairs of the study place. In addition, checked for applicability in terms of the need for finance, material and other issues to execute each steps). As described in detail in previous study done in quite different part/region of Ethiopia [20], here in this study CPPs were approached and provided with informed consent with the intent to confirm their agreement to participate. As soon as we received their consent, they were given a sample b2 agonist ‘‘Salbutamol puff ‘‘, which is the most common MDI in the study area and requested to exhibit the technique right to the interviewer considering as if he/she is asthmatic patient looking for medication use. The interviewer was one of the investigator acting as a simulated medication user but the participants already knew as he is not a real patient. Along with the interviewer, one of the very senior, experienced pharmacist and principal investigator stood up aside in the pharmacy/drugstore and watched out carefully the dispensers skill and knowledge while they are demonstrating then finally gave score for the given 11 criteria (Table 1) immediately at the Pharmacy or Drug store but the score given kept secretly (never disclosed to the pharmacist or druggist under evaluation). Scores were categorized as correctly, incorrectly demonstrated and skipped steps. According to NAEPP, CPPs’ in inhalational techniques was demonstrated based on their capability to exhibit all the essential steps (step one, two, five, six, seven and eight) and a total score of 7 or above and those who were not demonstrating all the essential steps correctly and scores <7 were considered as having poor competency in demonstrating inhalational technique.

**Table 1:** Frequency of evaluation of respondents to demonstrate each step of metered dose inhaler technique, 2017.

Variables	Correct	Incorrect	Skipped
1*Shake the contents well	45(72.6)	2(3.2)	15(24.2)
2*Remove the cap	50(80.6)	2(3.2)	10(16.1)
3. Hold the inhaler upright	32(51.6)	10(16.1)	20(32.3)
4.Tilt the head back slightly	6(9.7)	9(14.5)	47(75.8)
5*Breath out slowly	29(46.8)	3(4.8)	30(48.4)
6*Open mouth with inhaler 1 to 2 inches away or in the mouth with the lips tightly sealed around it	23(37.1)	10(16.1)	29(46.8)
7*Begin breath in slowly and deeply through the mouth and actuate the canister once	14(22.6)	3(4.8)	45(72.6)
8*Hold breath for 10–20 sec	18(29.0)	9(14.5)	35(56.5)
9.Exhale and wait one minute before the second dose	16(25.8)	5(8.1)	41(66.1)
10.Shake again before the second dose	26(41.9)	2(3.2)	34(54.8)
11 After use, replace the mouth piece cover	40(64.5)	2(3.2)	20(32.3)

*essential steps according to NAEPP.

### Data entry and analysis

Data was edited; cleaned, coded, entered, and then analyzed using Statistical Package for Social Studies (SPSS) version 20 for Windows. Categorical data were described in frequencies and percentages. Practice values were categorized by data transformation tool into two groups as poor and adequate based on the final scores. Chi-square test of association was done to point out factors associated with competency in MDI technique but we took the fisher exact test to show predictors in the table since our data did not fulfill the assumptions for Pearson's chi-square.

### Ethical considerations

The study was ethically approved by the Institutional Review Committee of School of Pharmacy, University of Gondar with an approval number of UoG-SoP-130/2017. The data collected was kept anonymous and recorded in such a way that the involved pharmacy professionals could not be known. Moreover, the evaluation result had not been disclosed to pharmacy professionals under evaluation.

### Operational definitions

In our study;

#### Pharmacy

It represents a drug shop having the mandate to hold any medicine and medical equipment. In addition, the professional who is supposed to dispense inside the pharmacy is ‘A pharmacist’ no one else is allowed to dispense according to the (Food, Medicine, Healthcare Administration and Control Authority (FMHACA) of Ethiopia.

#### Drug store

Unlike Pharmacy, Drug store is a drug shop but the medicine to be dispensed here is restricted that means it is not legal to hold every medications in this medicine retail outlet. For instance: It is not allowed to hold medications like psychotropic/narcotic drugs. In addition, the professional who is supposed to dispense inside the drug store is ‘A druggist ‘.

#### Pharmacists

In Ethiopia, Those are professionals having bachelor degree from private or Government University. In addition, they took all the courses that one medication expert has to know at the end. They took course for 4 years and now a days for five years.

#### Druggists

We can use this name interchangeably with’ **Pharmacy technician’.** In Ethiopia those are professionals having **‘diploma degree”** from college, it is not university level. They took course**s** for 3 years only and it is not that much comprehensive like pharmacists.

## Results

Among 70 community pharmacy professionals approached, 62 (32 pharmacists and 30 druggists) completed the survey with a response rate of 85.6%. Eight community pharmacy professionals were not willing to involve in the study due to different reasons like; being busy and refusal to respond. Among the respondents, 39 (62.9%) were males with a mean age of 33.9 with a standard deviation of ±10.05 years. The Majority of respondents 43 (69.4%) had a work experience of <5 years. The detailed demographics of respondents are shown in (Table 2).

**Table 2:** Socio-demographic characteristics of the respondents, Gondar, 2017 (N = 62).

Variable		Frequency (%)
Gender	Male	39(62.9)
	Female	23(37.1)
Age(years)	20–29	30(48.4)
	30–39	17(27.4)
	40–49	10(16.1)
	≥ 50	5(8.1)
Qualification	Druggist	30(48.4)
	Pharmacist	32(51.6)
Work experience	<5years	43(69.4)
	>5years	19(30.6)
Salary per month (birr)	<1499	8(12.9)
	1499–3000	31(50.0)
	>3000	9(14.5)
	Owner	14(22.6)
Working sector	Drug store	20(32.3)
	Pharmacy	42(67.7)
Training on MDI use	Yes	5(8.1)
	No	57(91.9)

All steps are adapted from NAEPP.

Out of 62 participants, only 5 got training on MDI use. In this study, only three (4.8%) respondents were competent enough to demonstrate the essential steps correctly.

Respondents were evaluated on their competence of MDI technique. Accordingly, step 2 (removing the cap) was the most frequently demonstrated (80.6%) step followed by step 1 (shake the content well) (72.6%). On the other hand, step 4(tilt the head back slightly) and step 7(begin breath in slowly and deeply through the mouth and actuate the canister once) were among the least demonstrated steps (9.7% and 22.6%, respectively). The details of the frequency of respondents to demonstrate each MDI technique steps described below at (Table 1).

**Fig 1:**
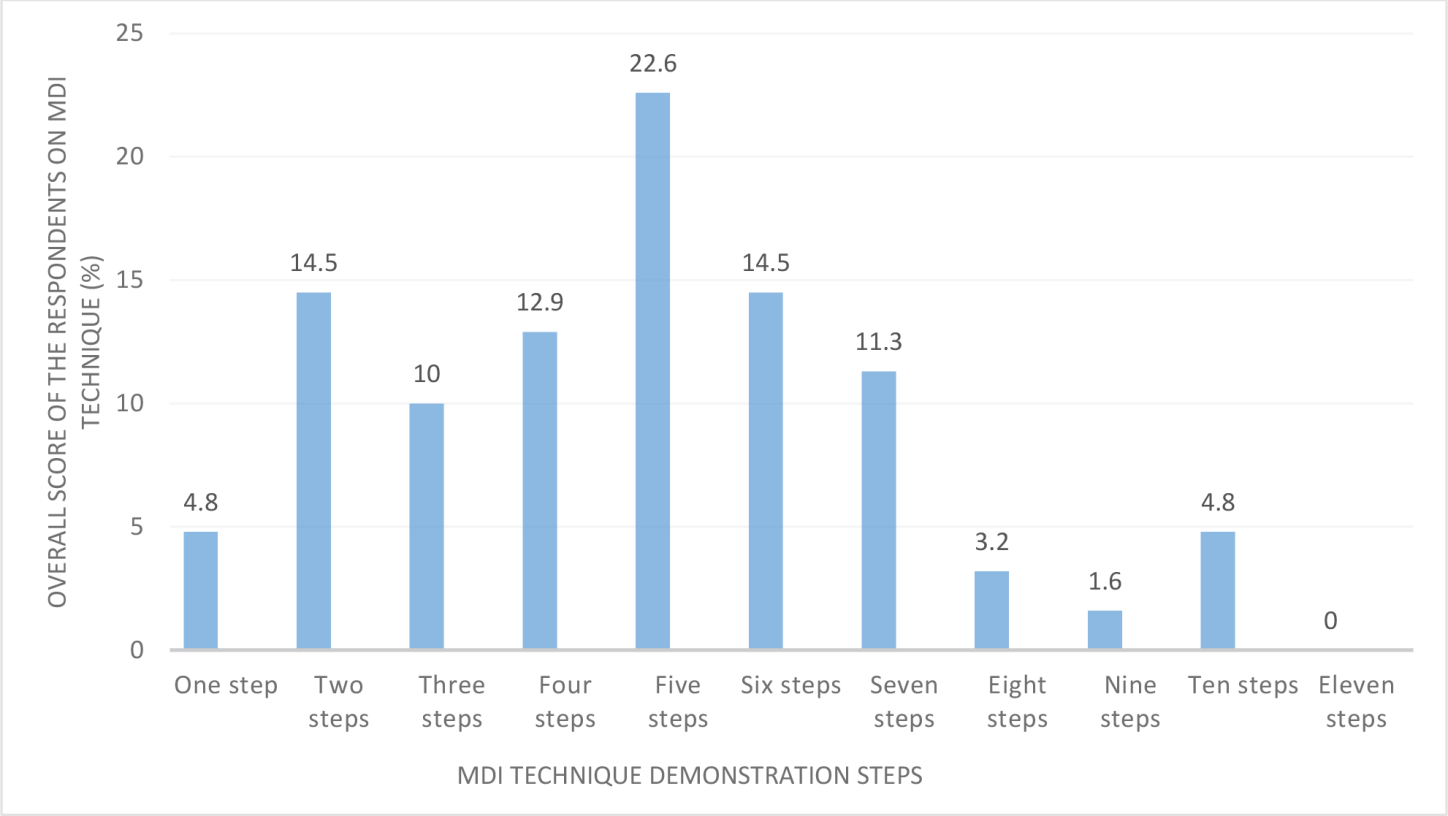
Overall score of the respondents on MDI technique, Gondar, 2017.

This study revealed that only 13 participants got score seven or above, but most of them missed the pertinent steps which included steps 1, 2, 5, 6, 7 and 8(Fig 1).

Fisher exact test revealed that there was a significant difference (*P* = 0.015) in competency of demonstrating adequate inhalational technique among respondents who took training on basic inhalational techniques and who did not. Unlike training, other variables such as educational status, work experience, working sector had no significant association with competency of delivering sufficient inhalational techniques (Table 3).

**Table 3:** Fisher exact test to explore potential predictors of competency of adequate inhalational technique, 2017.

Variables	Adequacy of MDI technique		*p-*value
	Competent	Incompetent	
Education level			
Druggist	1	29	0.525
Pharmacist	2	30	
Work experience			
< 5 years	1	42	0.220
>5 years	2	17	
CDROs			
Drug store	1	19	0.696
Pharmacy	2	40	
Training on MDI technique			
Yes	2	3	0.015*
No	1	56	
Gender			
Male	1	38	0.308
Female	2	21	

*Significant at *P*<0.05.

## Discussion

The changing impact of community pharmacy professionals from their traditional dispensing responsibilities to a greater contribution to population health is being accepted in the entire world [24]. To the best of our knowledge, this is the pioneer survey to explore the competency of community pharmacy professionals in MDI technique in northwest Ethiopia. In this study, the community pharmacy professionals' demonstration of correct use of MDI was poor. The poor demonstration seen in this study concurs to findings from other countries that reported a high proportion of Pharmacy professionals showing inappropriate use of inhaler [25]. One study revealed that poor inhaler use techniques among asthmatic patients has been justified as it was because of the insufficient education of patients by the health care providers [26].

A recent study suggested around 25% of patients had not received any verbal instructions for the use of their prescribed inhaler [27]. When given, instructions were often hurried, of poor quality and not reinforced. Merely an estimated 11% of patients received follow-up assessment and education about their device use techniques [28].

A study concluded that training on basic MDI use techniques could improve the skills of patients and providers [14]. In our study, very poor performance towards MDI use techniques among CPPs was observed, this could be partially explained by the very poor pharmacy education curriculum of the country which focuses mainly on theoretical topics than practical sessions on the real society. In addition, lack of frequent supervision of CPPs performance so as to take appropriate interventions might be the reason for the poor competency of CPPs in correctly demonstrating MDI to their clients. As to the findings of this study, only 13 out of 62 participants involved in survey got score seven or above.

Yet, most of them missed the essential steps (1, 2, 5, 6, 7, and 8) with only three professionals capable of addressing the essential steps and no one got all steps right in this study unlike the finding from Oman where 15% of the respondents performed all the steps correctly [29]. The study conducted in Mekelle reported that only two respondents had adequate competency in demonstrating MDI [20].

It is perhaps not surprising that patients frequently use their device(s) wrongly since healthcare professionals' understanding of the proper use of these devices is also poor. A recent study conducted in the UK stressed that only 7% of healthcare providers, including pharmacists, could demonstrate all the accurate steps in MDI use [12].

Several studies uncovered similar results with doctors, nurses and pharmacists having poor knowledge on the optimal use of different inhalers. It is essential that those providing patient training are themselves capable of showing steps correctly [27]. Among essential steps, begin breath in slowly and actuate the canister once, were the most skipped step in our findings, which is quite comparable to the Ethiopian study in Mekele [20]. However, in the Nigerian study, this step was demonstrated usually by the practicing pharmacists (90.2%) [19]. While a study conducted in Iran revealed that “depressing the canister” was the repeatedly occurring error [3]. From all steps, the frequently skipped and/or incorrectly responded was “tilt the head back” unlike the study done in Mekelle [20], where Exhale & wait one minute before the second dose was the frequently skipped and incorrectly demonstrated step. Similar result was also observed from the study done in Nepal [18].

As to the fisher exact test there was significant association (*P* = 0.015) in competency of demonstrating adequate inhalational technique among respondents who took training on basic inhalational techniques in Gondar town health office and who did not. However, this significance seen in our study could be partially resulted from the small number of trained pharmacy professionals. To the contrary, gender influence on their competency was found to be insignificant which is similar with the result of a study done by Chafin et al [30].

## Strength and limitation

This survey highlights an area of community pharmacy practice where there is lack of literature in Ethiopia. Yet, the survey has some limitations that should be noted while interpreting the results. As far the study was a cross-sectional survey conducted in Gondar town, caution should be exercised when generalizing to other cities and regions in Ethiopia. Moreover, our direct visit of community pharmacy professionals at their work place could affect the responses as it may be subjected to respondent bias, which could have been reduced had our study been simulated patient approach. Even with the above limitations, this survey has significant implications for improving the active engagement of community pharmacy professionals in health promotion and diseases state management for patients with asthma by using the result of this study as input to recommend the town health office to arrange a regular capacity building session to CPPs with the intent to improve their knowledge and skills towards MDI use techniques.

## Conclusion

Community pharmacy professionals' competency of MDI use technique was poor. Despite involvement of all participants in patient counseling on inhalers, none of them were able to execute all steps correctly, which shows that patients who had the chance to visit those CPPs in the town were not adequately instructed. In our study, significant association was not found between educational status, work experience and work sector with competence of MDI technique. However, significant difference (*P* = 0.015) noted in competency of demonstrating adequate inhalational technique among respondents who took training on basic inhalational techniques and who did not, having in mind that this significant difference might be as a result of low number of trained participants.

## Implications

To strongly integrate community pharmacies into the future asthma care and optimize the contribution of pharmacy professionals, interventions like establishing and providing regular capacity building education to CPPs has to be in action by all stakeholders. Follow-up studies seeking community pharmacy professionals' involvement through utilizing mixed studies including cross-sectional and simulated patient methodology may also be needed nationally to identify barriers and to better inform regulatory bodies.

## Supporting information

S1 Dataset Spss of MDI.sav. (SAV)

## Abbreviations

COPD: chronic obstructive pulmonary disease
MDI: metered dose inhaler
NRAD: National Review of Asthma Deaths
CPP: Community Pharmacy professionals
NAEPP: National Asthma Education and Prevention Programs of America
SPSS: Statistical Package for the Social Sciences
